# The SP‐TLR axis, which locally primes the nasal mucosa, is impeded in patients with allergic rhinitis

**DOI:** 10.1002/clt2.12009

**Published:** 2021-03-24

**Authors:** Olivia Larsson, Ola Sunnergren, Claus Bachert, Susanna Kumlien Georén, Lars Olaf Cardell

**Affiliations:** ^1^ Division of ENT Diseases Department of Clinical Sciences, Intervention and Technology Karolinska Institutet Stockholm Sweden; ^2^ Department of Otorhinolaryngology Ryhov County Hospital Jönköping Sweden; ^3^ Upper Airways Research Laboratory Ghent University Ghent Belgium; ^4^ Department of ENT Diseases Karolinska University Hospital Stockholm Sweden

**Keywords:** allergic rhinitis, neutral endopeptidase, NK1, substance P, TLR4

## Abstract

**Background:**

Substance P (SP) and toll‐like receptors (TLRs) contribute to airway disease, particularly during viral infection. We recently demonstrated that SP can act as an initial response to viral stimuli in the upper airway by upregulating TLRs in the nasal epithelia (the SP‐TLR axis). Patients with allergic rhinitis (AR) suffer from prolonged airway infections. The aim of the present study was to examine if patients with AR exhibit a disturbance in the SP‐TLR axis.

**Method:**

Human nasal biopsies and human nasal epithelial cells (HNEC) from healthy volunteers and patients with AR were cultured in the presence of SP. Epithelial expression of TLR4, neutral endopeptidase (NEP) and neurokinin 1 (NK1) were evaluated with flow cytometry and/or quantitative polymerase chain reaction after 30 min to 24 h. The effect of SP on nasal lipopolysaccharide‐induced interleukin‐8 (IL‐8) release was investigated.

**Results:**

SP stimulation of tissue from healthy volunteers resulted in a transient increase of the TLR4 expression, whereas stimulation of AR patient‐derived material led to a delayed and prolonged upregulation of TLR4. NEP expression in HNEC was lower in AR than healthy controls whereas NK1 receptor expression was increased. SP pretreatment increased TLR4‐dependent IL‐8 expression in healthy controls, but not in AR.

**Conclusions:**

SP‐induced regulation of TLR4 in the human nasal mucosa is disturbed in AR. An altered SP‐mediated innate immune response may contribute to the dysfunctional and often prolonged responses to infection in AR.

## 1 BACKGROUND

Allergic rhinitis (AR) is characterised by seasonal or persistent inflammation in the upper airways, leading to rhinorrhoea, nasal congestion, itching, and sneezing. It is a well‐founded clinical observation that patients with AR often are more prone to develop infectious airway disease than nonallergic individuals. This includes sinusitis, as well as otitis media with effusion. The close association of nasal allergies with these conditions has been supported by extensive epidemiological evidence.^1^ Many studies have also highlighted alterations in the innate immune response in atopic airway diseases, leading to susceptibility to infections.^2^, ^3^ In particular, expression levels and function of toll‐like receptors (TLRs), as early responders to viral and bacterial particles, have been shown to be altered.^4^, ^5^, ^6^, ^7^

Substance P (SP) is known to be locally induced upon nasal allergen challenge^8^ and its upregulation in symptomatic AR is well‐documented.^9^ As a mediator of neurogenic inflammation, SP induces vasodilation, vascular leakage, mucous secretion, and inflammatory cell recruitment, all contributing to the hallmark symptoms of AR.^10^ In addition to its role in neurogenic inflammation, recent studies have highlighted a function for SP as mediator of innate immunity. SP has been shown to contribute to the development of an antiviral innate immune response in mice,^11^ as well as regulate messenger RNA (mRNA) levels of TLRs in human mast cells.^12^ We recently published a study outlining a novel role for SP as a regulator of the innate immune system in the nasal mucosa. SP, released following stimulation with the viral mimetic and TLR7 agonist R837 induced a rapid increase in the expression of several epithelial TLRs including TLR3 and TLR4.^13^ Hence, it is tempting to conclude that this SP‐TLR axis primes the nasal mucosa for incoming infections.

The action of SP is known to be controlled by a number of mechanisms. First, SP is broken down by a variety of peptidases, most notably neutral endopeptidase (NEP).^14^ NEP exists in a soluble and membrane‐bound form and is expressed on epithelial cells, vessels and serous cells of submucosal glands of the nasal mucosa.^15^ NEP is released within 20 min after a nasal allergen challenge,^15^ and it is known to limit the concentration of SP that reaches its receptor as inhibition of NEP potentiates the action of SP in the human nasal mucosa.^16^ Second, that action of SP is controlled by its cognate receptor neurokinin 1 (NK1). Increased expression of NK‐1, as seen in asthmatic airways,^17^ increases the effect of SP. However, upon SP‐NK‐1 interaction, the agonist‐receptor complex is internalized, limiting further and continued action of SP.^18^

As AR is associated with alterations in the expression of SP, the present study was designed to compare elements in SP‐TLR axis in nasal biopsies and epithelial cells derived from patients with AR with corresponding material from healthy individuals.

## 2 METHODS

### 2.1 Ethics statement

The study was approved by the local Ethical Committee, Stockholm, Sweden (number 2014/299 and 2016/826). All participants gave their written, informed consent. All procedures were conducted according to the principles expressed in the Declaration of Helsinki.

### 2.2 Subjects and study design

The study included 30 nonsmoking, non‐astmatic, patients with moderate to severe birch and/or grass pollen‐induced seasonal AR, and 35 healthy volunteers. Human nasal epithelial cells (HNEC) were isolated from 24 healthy subjects and 22 AR patients; nasal biopsies were isolated from 11 healthy subjects and 8 AR patients. All samples were acquired outside the pollen season. Patient characteristics are listed in Table 1.

**TABLE 1:** Patient characteristics

	Number	Age (mean ± *SEM*)	M:F ratio
HNEC			
Healthy control	24	33.7 ± 1.89	7:17
Allergic rhinitis	22	31.9 ± 1.68	10:12
Biopsies			
Healthy control	11	25.1 ± 1.21	4:7
Allergic rhinitis	8	27.4 ± 2.03	3:5

Abbreviation: HNEC, human nasal epithelial cell.

The diagnosis of birch or grass pollen‐induced AR was based on a positive history of seasonal AR as well as a positive ImmunoCap Rapid (Asthma/Rhinitis Adult) test (Phadia; Thermo Fisher Scientific). Patients who did not meet both criteria were excluded from the study. The ImmunoCap Rapid (Asthma/Rhinitis Adult) assesses the presence of circulating specific immunoglobulin E against 10 common airborne allergens, including pollen (birch, timothy, mugwort, olive, wall pellitory), house dust mites (HDM), mould and common animal allergens (cat, dog, cockroach). Exclusion criteria included a history of upper airway infection within 2 weeks prior to visit, treatment with local or systemic corticosteroids within 2 months before the visit, and history of chronic rhinosinusitis with or without presence of nasal polyposis.

Five AR patients tested positive for allergy towards common animals allergens and one AR patient tested positive for allergy towards HDM. Two AR patients had comorbid allergic asthma.

### 2.3 Isolation and culture of HNECs

HNECs were isolated by nasal brushing as described previously.^13^ Cells for stimulation experiments were maintained in collagen‐coated tissue culture flasks in Keratinocyte Serum‐Free Medium (KSFM; Gibco), supplemented with 0.005 µg/ml epidermal growth factor (Gibco), 0.05 mg/ml bovine pituitary extract (Gibco), 40 U/ml penicillin, 40 µg/ml streptomycin and 0.025 µg/ml Fungizone (complete KSFM). All cells were cultured in a humidified chamber at 37°C with 5% CO_2_. Cells from passages 2–5 were used, and all were positive for EpCAM (>90%), an epithelial‐specific adhesion molecule.

### 2.4 Isolation and culture of human nasal biopsies

Human nasal biopsies (HNBs) were isolated as described previously.^19^ Two biopsies per patient were obtained from the inferior turbinate following topical application of local anaesthesia containing lidocaine hydrochloride/nafazoline (24 and 0.17 mg/ml) for 20 min. Following extraction, biopsies were immediately placed in warm complete KSFM and incubated overnight in a humidified chamber at 37°C with 5% CO_2_.

### 2.5 Epithelial cell stimulation

HNEC were seeded onto 24‐well culture plates (200,000 cells/well) in 1 ml complete KSFM and incubated overnight. Cells were subsequently stimulated with 30 µg/ml R837 (InvivoGen) for 15, 30, 60 or 240 min or 30 nM SP (Sigma‐Aldrich) for 30, 60 or 240 min. Cell culture supernatants were collected and stored at −80°C until further use. Cells were assessed for expression of TLR4, NEP or NK1R with flow cytometry or mRNA expression of TLR4 with quantitative polymerase chain reaction (qPCR).

### 2.6 Biopsy stimulation

Biopsies were dissected into four equal pieces and placed into 48‐well culture plates in 500 μl complete KSFM, where they were allowed to equilibrate for 30 min prior to stimulation. Biopsies were subsequently stimulated with 30 µg/ml R837 for 15, 30, 60 or 240 min or 30 nM SP for 30, 60 or 240 min. Cell culture supernatants were collected and stored at −80°C until further use. Biopsies were placed through a 100 μm cell strainer (BD Falcon) into complete KSFM. The cells were washed and centrifuged, after which the supernatant was aspirated and discarded. Cells were subsequently assessed for expression of TLR4, TLR7, NEP and NK1R with flow cytometry.

### 2.7 Flow cytometry

For detection of EpCAM, TLR4, TLR7, NEP and NK1R, epithelial cells and biopsies were stained with the following antibodies: EpCAM‐fluoroscein‐isothiocyanate (347197; BD Bioscience), TLR4‐AlexaFluor700 (FAB6248N; RnD Systems), TLR7‐phycoerythrin (IC5875P; RnD Systems), CD10‐PE‐Cy7 (565282; BD Bioscience) and NK1R‐allophycocyanin (FAB66871A; RnD Systems). Cells were stained with aqua LIVE/DEAD® Fixable dead stain (Thermo Fisher Scientific) to detect dead cells; Fc Block (BD) was used to block nonspecific binding. Fluorescence minus one controls were used to assess background fluorescence levels. The IntraPrep Permeabilisation Reagent kit (Immmunotech; Beckman Coulter) was used to detect intracellular TLRs. Cells were analysed on an LSR Fortessa Flow Cytomter (BD Biosciences). Events in the range of 5000–30,000 were recorded. Data were analysed with FlowJo Analysis Software (TreeStar).

### 2.8 RNA extraction and RT‐PCR

Epithelial cells were lysed with RLT Buffer (Qiagen) supplemented with 40 mM dithiothreitol. Total RNA was extracted using the RNeasy Micro Kit Plus (Qiagen); quantity and quality of RNA was determined by spectrophotometer using the wavelength absorption ratios 260/280 and 260/230. Four hundred nanograms RNA was reverse transcribed into cDNA using the Omniscript Reverse Transcription Kit (Qiagen) with Oligo (dT)_18_ primers (Thermo Fisher Scientific) on a xPeltier Thermal Cykler 200 (MJ Research).

### 2.9 Multiplex quantitative PCR

Real‐time multiplex qPCR was performed using a dual‐labelled probe technique with the Quantifast Multiplex PCR + R kit (Qiagen) on a Stratagene Mx3000P Cycler (Agilent Technologies). All reactions were run with the following program: activation at 95°C for 5 min, followed by 40 cycles of two‐step cycling at 95°C for 45 s and 60°C for 45 s. Primers and probes for reference genes (*actb, gapdh*) and target genes (*tlr4*) were acquired from Sigma‐Aldrich and are detailed in Table 2. Taqman primers for *cxcl8* (Hs00174103_m1) were acquired from Applied Biosystems. Analysis of *C*_t_ values was performed using the ΔΔ*C*_t_ method.

**TABLE 2:** qPCR primers and probes

	Sequence (5ʹ–3ʹ)
GAPDH	
Forward primer	ACAACGAATTTGGCTACAGC
Reverse primer	AGTGAGGGTCTCTCTCTTCC
Probe	ACCACCAGCCCCAGCAAGAGCACAA
ACTB	
Forward primer	CAAGATGAGATTGGCATGGC
Reverse primer	CACATTGTGAACTTTGGGGG
Probe	TGACAGCAGTCGGTTGGAGCGAGCA
TLR4	
Forward primer	CAGAGTTTCCTGCAATGGATCAAG
Reverse primer	TGCTTATCTGAAGGTGTTGCACAT
Probe	AGAGGCAGCTCTTGGTGGAAGTTGAACGA

Abbreviation: qPCR, quantitative polymerase chain reaction.

### 2.10 Measurement of SP

Levels of SP from cell or biopsy culture supernatants were measured with an SP ELISA kit (Cayman Chemical), according to the manufacturer's instructions.

### 2.11 Statistical analysis

Data were analysed with GraphPad Prism software (Version 6.0). Results are expressed as mean ± *SEM*. In all experiments, *n* is equal to the number of subjects. Analysis was performed using a two‐way analysis of variance, followed by a Fisher's posttest. A *p* value of 0.05 or less was considered statistically significant.

## 3 RESULTS

### 3.1 R837 induces SP release in the nasal mucosa

To ascertain if the previously demonstrated R837‐induced SP release in nasal mucosa was affected by the allergic condition per se*,* HNBs and HNECs from healthy volunteers and patients with AR were stimulated with R837. SP release was unaffected by the allergic versus non‐allergic status, neither in the HNB specimens (*p* = 0.9), nor in the HNEC material (*p* = 0.07; Figure S1).

### 3.2 The SP‐induced TLR4 upregulation in the nasal mucosa is delayed in AR

**FIGURE 1:**
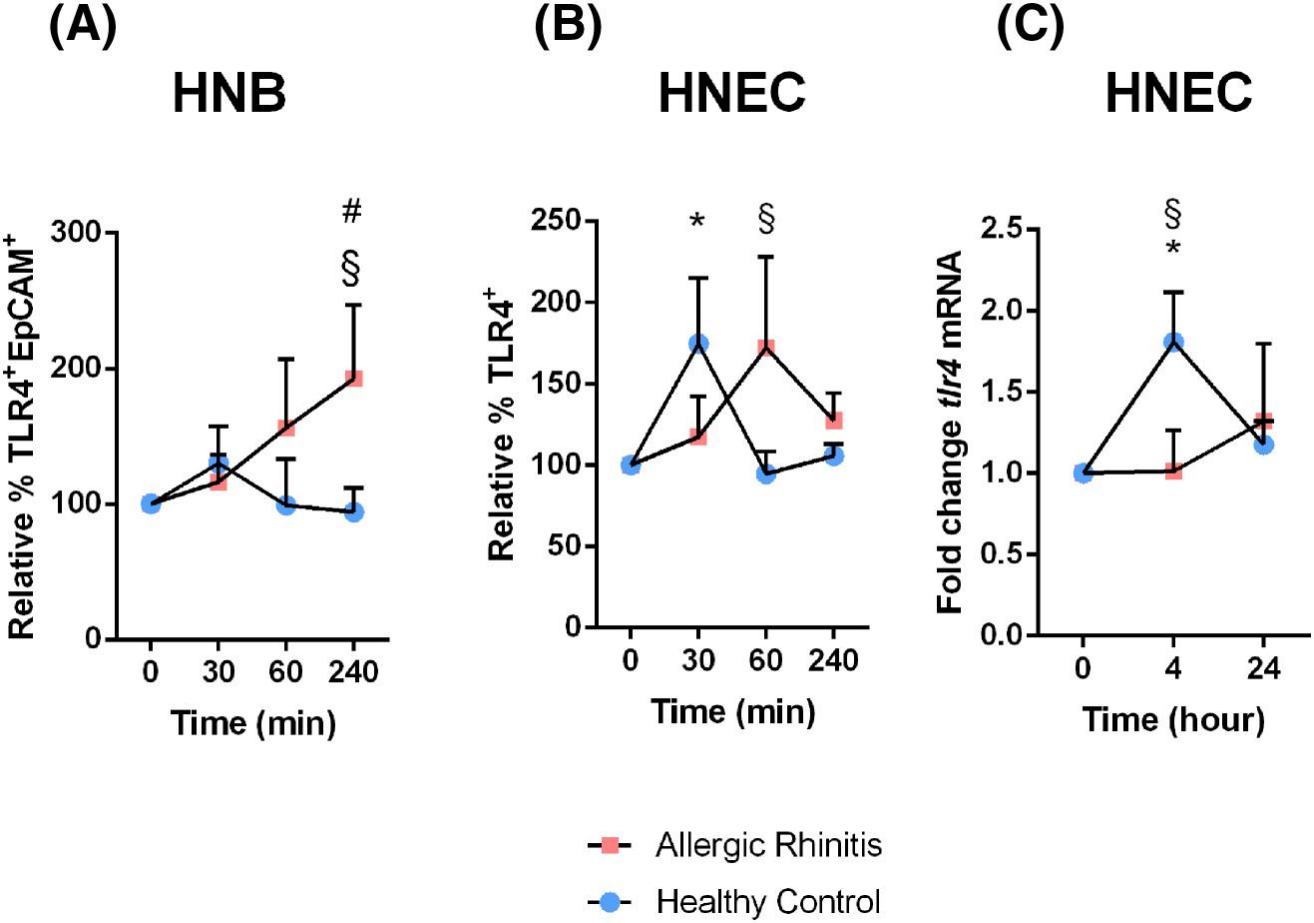
The effect of SP on epithelial TLR4 expression. (A) HNB and (B) HNEC were stimulated with SP (30 nM). The change in TLR4 expression was assessed at 30, 60 and 240 min with flow cytometry. In addition, (C) HNECs were also evaluated on an mRNA level after 4 and 24 h of stimulation with SP (30 nM). Values are presented as mean percental change in relation to control TLR expression ± *SEM* (A and B) or mean fold change of mRNA ± *SEM* (C). (A) *n* = 7–8; (B) *n* = 7–10; (C) *n* = 4–6. *p < 0.05 comparing specified time points to ‘0’ in health volunteers, ^#^ *p* < 0.05 comparing specified time points to ‘0’ in AR patients, ^§^ *p* < 0.05 comparing healthy volunteers to AR patients at specified time points, using two‐way ANOVA with Fisher's posttest. ANOVA, analysis of variance; AR, allergic rhinitis; HNB, human nasal biopsies; HNEC, human nasal epithelial cell; mRNA, messenger RNA; SP, substance P; TLR4, toll‐like receptor 4

As no differences in SP release between healthy and AR patients were evident, the second arm of the two‐step SP‐TLR axis was investigated. HNBs and HNEC were stimulated with SP and TLR4 expression was assessed. Representative histograms of TLR4 expression in unstimulated HNB and cultured HNEC are presented in Figures S2 and S3. In healthy volunteers, SP stimulation resulted in a marked and transient increase in TLR4 protein in both specimens at 30 min, reaching significance for HNEC (*p* = 0.024). A similar increase was seen in AR samples, but with a later TLR4 peak (Figure 1A,B), demonstrating significant increases at 240 min in HNB (*p* = 0.047). TLR4 increases were significantly higher in HNB from AR patients at 240 min (*p* = 0.029) and in HNEC at 60 min (*p* = 0.046), as compared to respective material from healthy volunteers. Also, at the mRNA level, after 5–24 h, the TLR4 upregulation appeared earlier and was higher in HNECs from healthy individuals than in material from patients with AR (Figure 1C); at 4 hours *tlr4* mRNA was significantly upregulated in healthy controls (*p* = 0.016), and significantly elevated above values in AR patients (*p* = 0.032).

### 3.3 Decreased NEP expression in AR

**FIGURE 2:**
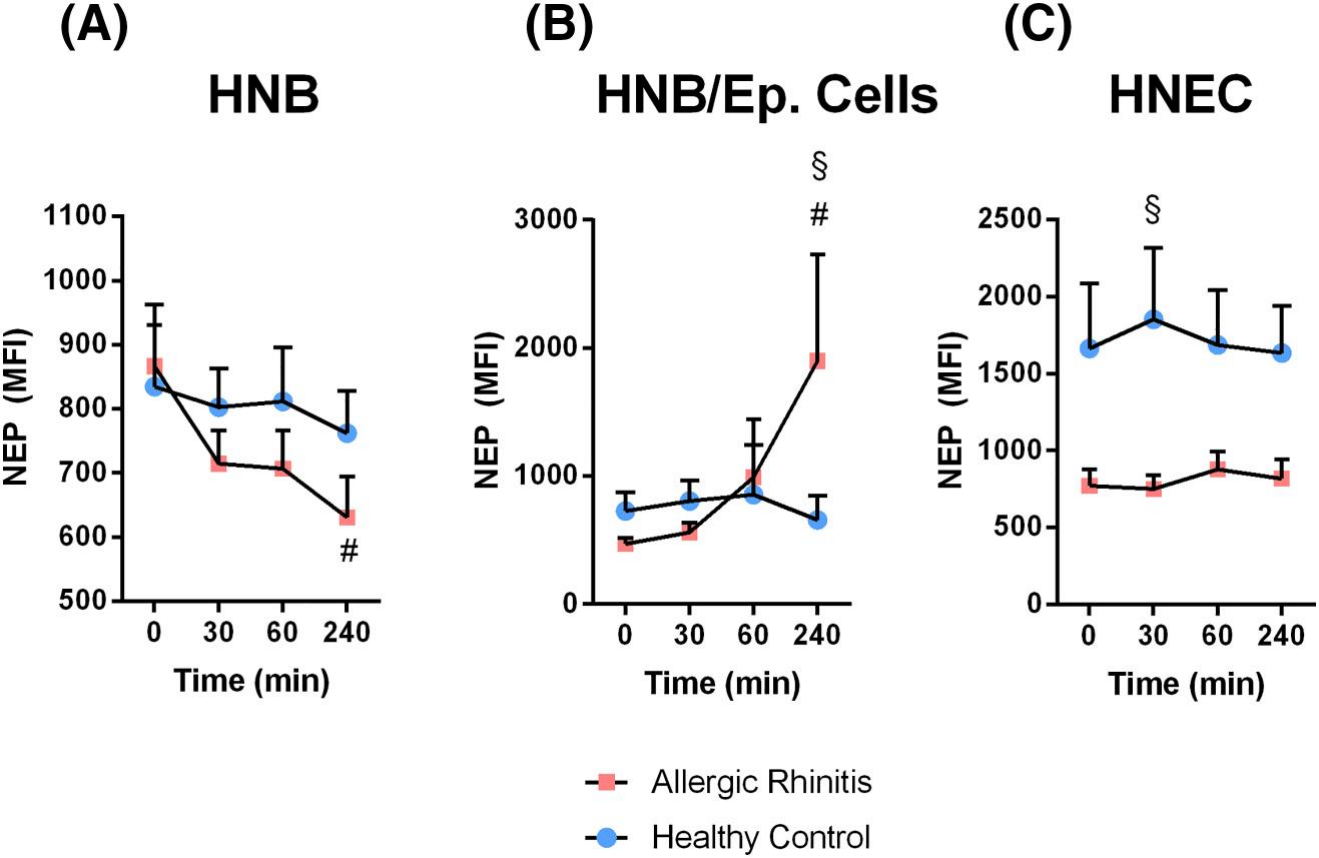
NEP levels before and after SP stimulation. Effect of SP stimulation on NEP expression in whole nasal biopsies (A), EpCAM^+^ epithelial cells from nasal biopsies (B) and or isolated and cultured HNEC (C). The baseline NEP expression was assessed directly from the obtained specimens/cells or after 30, 60 and 240 min stimulation with SP (30 nM) using flow cytometry. Values are presented mean MFI ± SEM. (A) *n* = 7–8; (B) *n* = 7–8; (C) *n* = 8–11, ^#^ *p* < 0.05 comparing specified time points to ‘0’ in AR patients, ^§^ *p* < 0.05 comparing healthy volunteers to AR patients at specified time points, using two‐way ANOVA with Fisher's posttest. ANOVA, analysis of variance; AR, allergic rhinitis; HNEC, human nasal epithelial cell; NEP, neutral endopeptidase; SP, substance P

NEP expression was found in HNEC and HNB (Figures S2 and S3). NEP levels were analysed in HNBs and HNEC during basal conditions as well as after stimulation with SP. No differences in baseline expression of NEP were evident in whole biopsies (Figure 2A, *p* = 0.7745). However, when only epithelial cells of the same biopsies were analysed, the NEP levels appeared to be lower in specimens from allergic patients (Figure 2B, *p* = 0.618). This difference was more marked, reaching significance (*p* = 0.0003) when the NEP analysis was repeated on the HNEC (Figure 2C). SP stimulation of the HNBs resulted in a decline in NEP expression in nasal biopsies. This reduction appeared to be more pronounced in biopsies from allergic individuals, reaching significance at 240 min (*p* = 0.04) (Figure 2A). SP stimulation had no effect on NEP expression in epithelial cells from nasal biopsies from healthy controls, whereas a marked increase in NEP was evident 240 min after stimulation in allergic individuals (*p* = 0.009; Figure 2B). The levels of NEP in HNEC seemed to be unaffected by SP, with the differences in levels observed during the basal conditions being maintained (Figure 2C).

### 3.4 Increased NK‐1 receptor expression in AR

**FIGURE 3:**
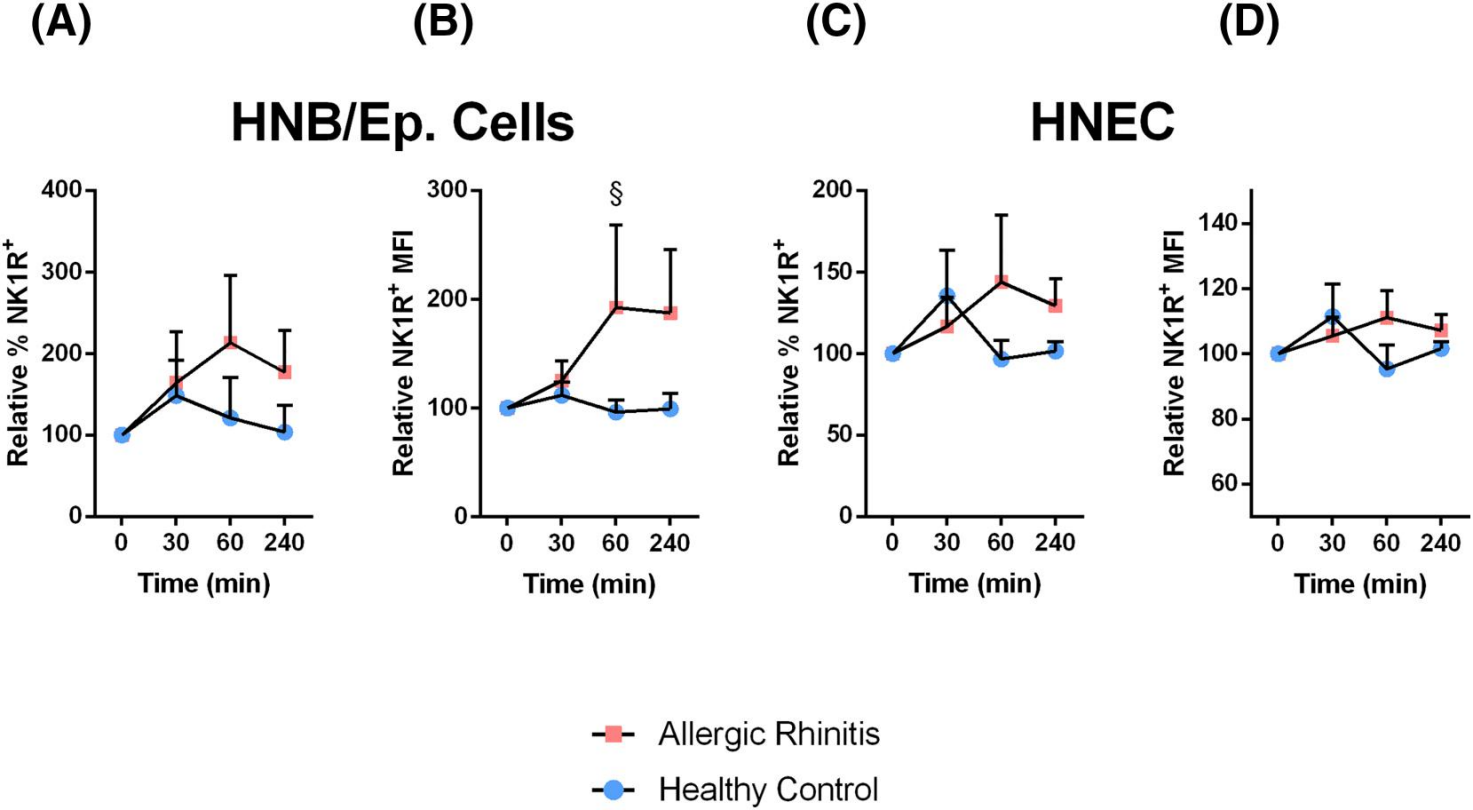
The effect of SP on NK1R receptor expression. (A and B) HNB and (C and D) HNEC were stimulated with SP (30 nM). The change in NK1R expression was assessed at 30, 60 and 240 min with flow cytometry. Values are presented as mean percental change in relation to control NK1R expression ± *SEM* (A and C) or mean MFI change in relation to control NK1R expression ± *SEM* (B and D). (A) *n* = 7; (B) *n* = 7–8; (C) *n* = 7–10; (D) *n* = 7–10. ^§^ *p* < 0.05 comparing healthy volunteers to AR patients at specified time points, using two‐way ANOVA with Fisher's post‐test. ANOVA, analysis of variance; AR, allergic rhinitis; HNB, human nasal biopsies; HNEC, human nasal epithelial cell; NEP, neutral endopeptidase; NK1R, NK‐1 receptors; SP, substance P

The effect of SP on TLR4 up‐regulation is mediated via NK‐1 receptors (NK1R) on the surface of epithelial cells.^13^ To assess whether the NK1R was affected in AR, its expression level was analysed with flow cytometry in HNBs and HNEC after stimulation with SP. Representative histograms of NK1 expression in unstimulated HNB and cultured HNEC are presented in Figures S2 and S3. SP caused a clear increase of the NK1R expression in epithelial cells in HNBs derived from patients with AR, particularly at 60 and 240 min, whereas the corresponding increase in healthy controls appeared to be limited and/or transient (Figure 3A,B). At 60 min, relative NK1R MFI was significantly higher in epithelial cells from AR HNB, as compared to respective material from healthy controls (*p* = 0.038). In HNEC, SP induced a trend towards an increase in the NK1R expression in cells from both healthy and allergic patients. However, the increases among the controls appeared earlier than in material from allergic volunteers (Figure 3C,D).

### 3.5 Pretreatment with SP does not prime lipopolysaccharide mediated IL‐8 release in AR

**FIGURE 4:**
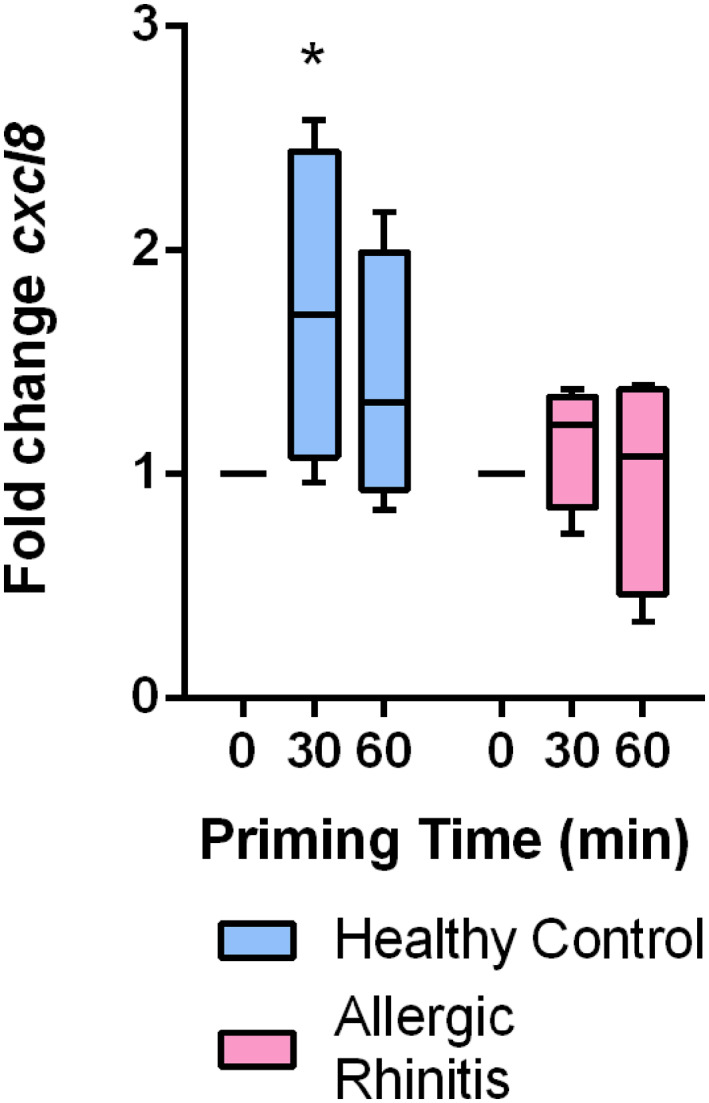
The effect of LPS on IL‐8 release in nasal mucosa pretreated with SP Cultured HNEC were stimulated with SP (30 nM) for 30 or 60 min, washed and subsequently stimulated with LPS (1 μg/ml) for 3 h. IL‐8 mRNA (cxcl8) levels were assessed with quantitative PCR. Values are presented as mean fold change ± *SEM*. *n* = 4, in both groups. *p < 0.05 comparing specified time points to ‘0’ in health volunteers using two‐way ANOVA with Fisher's post‐test. ANOVA, analysis of variance; HNEC, human nasal epithelial cell; IL‐8, interleukin‐8; LPS, lipopolysaccharide; mRNA, messenger RNA; PCR, polymerase chain reaction; SP, substance P

TLR4 is responsible for inducing early inflammatory responses against bacterial lipoproteins, such as lipopolysaccharide (LPS). In order to determine whether altered expression of TLR4 following SP stimulation resulted in altered responses to bacterial exposure, isolated HNEC were stimulated with SP for 30 or 60 min and then subsequently exposed to LPS. When the levels of IL‐8 mRNA were assessed 3 h after the LPS exposure, increases were evident in the control, nonallergic material, but failed to appear in cells from patients with AR (Figure 4).

## 4 DISCUSSION

The present study demonstrates that the ability for SP to regulate innate immunity is impaired in patients with AR, with a heightened and prolonged upregulation of TLR4, and an associated lack of expected increased response in LPS. These changes may be attributed to differences in the expression of NEP and NK1R, which are known to regulate expression and activity of SP. This finding is the first to show that SP may, at least in part, contribute to the more severe and prolonged infection‐induced diseases often seen in conjunction with airway allergy.

The present study demonstrates that SP rapidly, and transiently, upregulates nasal epithelial TLR4 in isolated cultures, as well as in a multicellular system, represented by nasal biopsies. This corroborates our previous findings^13^ and strengthens the existence of a neuropeptide‐TLR axis in the nasal mucosa. We additionally show that SP regulates expression of TLR4 on the mRNA level, a function of SP that has previously been demonstrated only in mast cells.^12^ It is unknown whether this is a direct action of SP or secondary to SP‐induced release of proinflammatory cytokines, known to upregulate TLR expression.^20^ Priming of epithelial cells with SP upregulated LPS‐induced transcription of the proinflammatory mediator IL‐8, a neutrophil chemoattractant. The ability for SP to rapidly prime LPS‐mediated immunity has been demonstrated previously; one study found that stimulation of dental pulp cells with SP for 30 min enhanced LPS‐induced NF‐κB binding activity, as well as LPS‐induced COX‐2 mRNA.^21^ These data suggest a multi‐faceted role for SP in rapidly initiating an innate immune response in the nasal mucosa.

SP is generally viewed as an instigator of inflammation; however, studies have demonstrated an additional ability for SP to regulate inflammation, through induction of IL‐10R and reduction of IFN‐γR.^21^ In line with this, the SP‐induced increase in TLR4 was transient in healthy individuals, returning to baseline after 60 min, suggesting control mechanisms are in place to prevent overactivation of the innate immune system by SP. Indeed, the action of SP is known to be regulated by a number of mechanisms, including the presence of soluble and membrane‐bound peptidases, such as NEP, and the internalization of the cognate SP receptor, NK1.

NEP is known to break down SP and limit its actions.^14^ As previously described,^15^ NEP was found to be expressed on epithelial cells in whole nasal biopsies, as well as other cells, likely to be serous cells of submucosal glands or endothelial and myoepithelial cells of small vessels. NEP expression was also present in isolated epithelial cells from nasal brushes, and levels stayed consistent upon culture. Previous studies have described the ability for SP to induce NEP,^22^ suggesting that SP can autoregulate through the release of its own peptidase. Indeed, SP stimulation of healthy nasal epithelial cells resulted in a transient increase in NEP after 30 min. This mimicked TLR4 expression, suggesting that transient increases in NEP and consequent decreases in SP may play a role in regulation of SP‐TLR axis. However, the same phenomenon was not seen in nasal biopsies, indicating that NEP may be of greater importance in local, epithelial regulation of SP function.

In contrast to NEP, regulation of epithelial NK1 expression in both nasal biopsies and isolated cultures, mimicked the pattern of SP‐induced TLR expression. In both culture systems, an increase in NK1 was evident after 30 min, after which levels decreased, indicative of receptor internalisation. SP‐induced TLR4 upregulation is NK1‐dependent^13^ and a decrease in the presence of NK1 would therefore directly influence TLR4 upregulation.

The main finding in this study was that the TLR4 response to SP in AR was significantly altered. In isolated epithelial cells, TLR4 upregulation was delayed, occurring at 60 min, compared to 30 min in healthy controls, after which levels decreased. More markedly, in a multi‐cellular system, epithelial TLR4 upregulation was sustained. These alterations were possibly associated with changes in the regulatory system of SP, namely expression of NEP and NK1. Indeed, in AR, baseline NEP expression in HNEC was found to be significantly downregulated, possibly a consequence of differing cytokine environments, as suggested by previous studies.^23^, ^24^ A decrease in NEP would inevitably result in increased levels of SP and altered responses of epithelial cells to SP.

Interestingly, whereas in healthy individuals SP stimulated NEP expression on epithelial cells on HNB correlated with TLR4 expression, the opposite was true in AR. Instead, SP‐stimulated NEP regulation in AR whole nasal biopsies correlated with changes in TLR4. Decrease of NEP in nasal biopsies was sustained and prolonged, mimicking and supporting the sustained increase in TLR4. Baseline levels of epithelial NEP were decreased in AR, indicating that SP expression, and subsequent TLR4 expression, may be controlled to a stronger degree by nonepithelial NEP. This difference may be one of the underlying reasons to disrupted TLR4 expression and function in AR.

Similar to healthy controls, SP‐induced epithelial NK1 regulation in AR mimicked that of TLR4, with delayed increases in HNEC and prolonged increases in epithelial cells from nasal biopsies. This further suggests that TLR4 and NK1 may be coregulated. Previous studies have documented that alterations in the structure of NK1 can alter the speed at which it is internalised. Truncated and glycosylated forms of NK1 internalise slower, leading to prolonged responses,^25^ To our knowledge, no studies have assessed if AR is associated with altered forms of NK1, and this is beyond the scope of this study. However, changes in NK1 structure may lead to prolonged presence on the cell‐surface, which would in turn prolong SP‐related responses on both TLR4 expression, and on typical SP‐induced inflammation.

Despite the delayed and prolonged increase in TLR4 in AR individuals, changes to LPS‐induced proinflammatory response were absent. Whereas in healthy individuals, SP upregulated transcription of *cxcl8* following LPS stimulation, no priming effect was evident in AR. The reason for this difference in function is unknown. One study has highlighted that upregulated TLR4 in allergy is associated with increased levels of Th2 and anti‐inflammatory cytokines.^5^ Therefore, sustained upregulation of TLR4 in allergy may lead to pronged release of pro‐allergy factors (e.g., IL‐13 or IL‐4), rather than typical proinflammatory cytokines.

Alterations in TLR4 and SP expression are well‐documented in AR. Levels of SP are consistently upregulated,^26^ which may in part be related to a decrease in epithelial NEP expression highlighted in this study. NK1 has also been shown to be upregulated in atopic airways.^17^ Most notably, TLR4 expression is upregulated in symptomatic and persistent AR,^5^, ^27^ in which levels of SP are known to be upregulated and constant, thus corroborating the continuous SP‐induced increase in TLR4 in vivo. However, despite increases in TLR4 expression, it is a well‐established clinical observation that patients with AR are more prone to develop infections and have an increased need for antibiotics,^1^ validating the inability for SP to prime the immune response in allergy.

It is worth pointing out a number of limitations to the study. First, the sample size is quite small (30 AR patients and 35 healthy donors), leading to large degree of variability in the results. A larger sample size would likely result in greater statistical stability of the data. Second, results from the study were based on in vitro stimulations; in vivo stimulations would be of interest to further confirm the differences in SP‐TLR axis in atopic and nonatopic individuals. Finally, only a subset of factors involved in the regulation of SP responses, namely NEP and NK1R, were investigated in this study. It is likely that other neuropeptides and inflammatory mediators are involved in this response. Further work on these mediators would offer a more complete picture of the SP‐TLR axis and would more intricately explain the differences seen in AR patients.

The present study demonstrates that in healthy individuals, SP functions as a regulator of innate immunity, particularly in upregulation TLR4 and thus priming LPS‐induced inflammation. In patients with AR this SP‐TLR axis appears to be impaired, with a heightened and prolonged upregulation of TLR4, associated with the lack of the expected increased response to LPS. This compromised ability for SP to regulate immunity may, at least in part, contribute to the more severe and prolonged infection‐induced diseases often seen in conjunction with airway allergy.

## AUTHOR CONTRIBUTIONS

Olivia Larsson, Susanna Kumlien Georén, Claus Bachert and Lars Olaf Cardell designed the experiments and interpreted the results. Ola Sunnergren recruited patients and took all biopsies. Olivia Larsson performed the experiments, analysed the data and wrote the paper. All authors read and approved the final manuscript.

## Supporting information

Supporting Information S1

Supporting Information S2

Supporting Information S3
