# Supplementary figures and images for: The SP‐TLR axis, which locally primes the nasal mucosa, is impeded in patients with allergic rhinitis
Source: Clin Transl Allergy. 2021 Mar 24;11(1):e12009. doi: 10.1002/clt2.12009 (PMC8099340; doi:10.1002/clt2.12009)

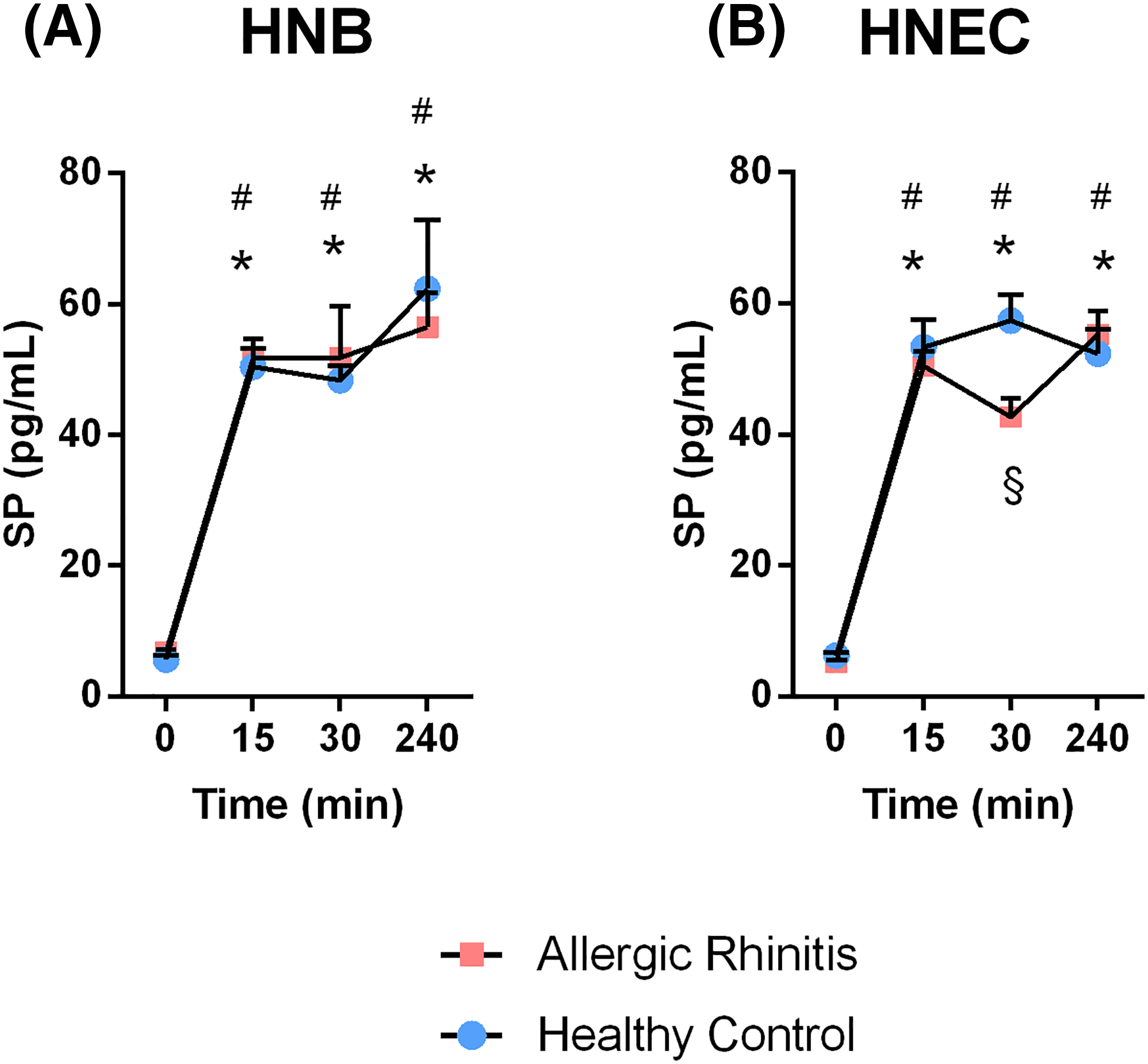

Supplement: Supplementary file 1 — Supporting Information S1 [file CLT2-11-e12009-s003.tif]

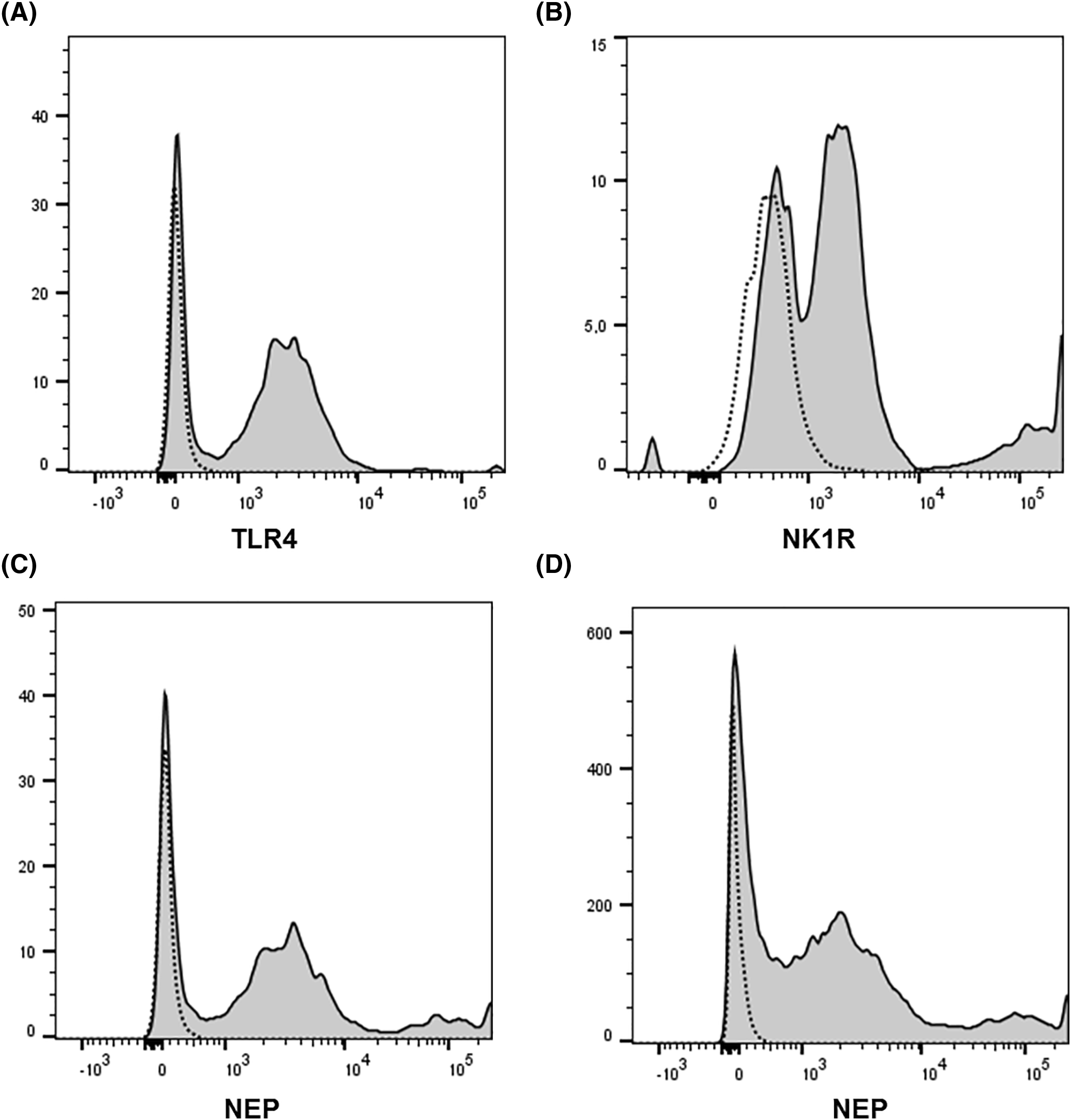

Supplement: Supplementary file 2 — Supporting Information S2 [file CLT2-11-e12009-s002.tif]

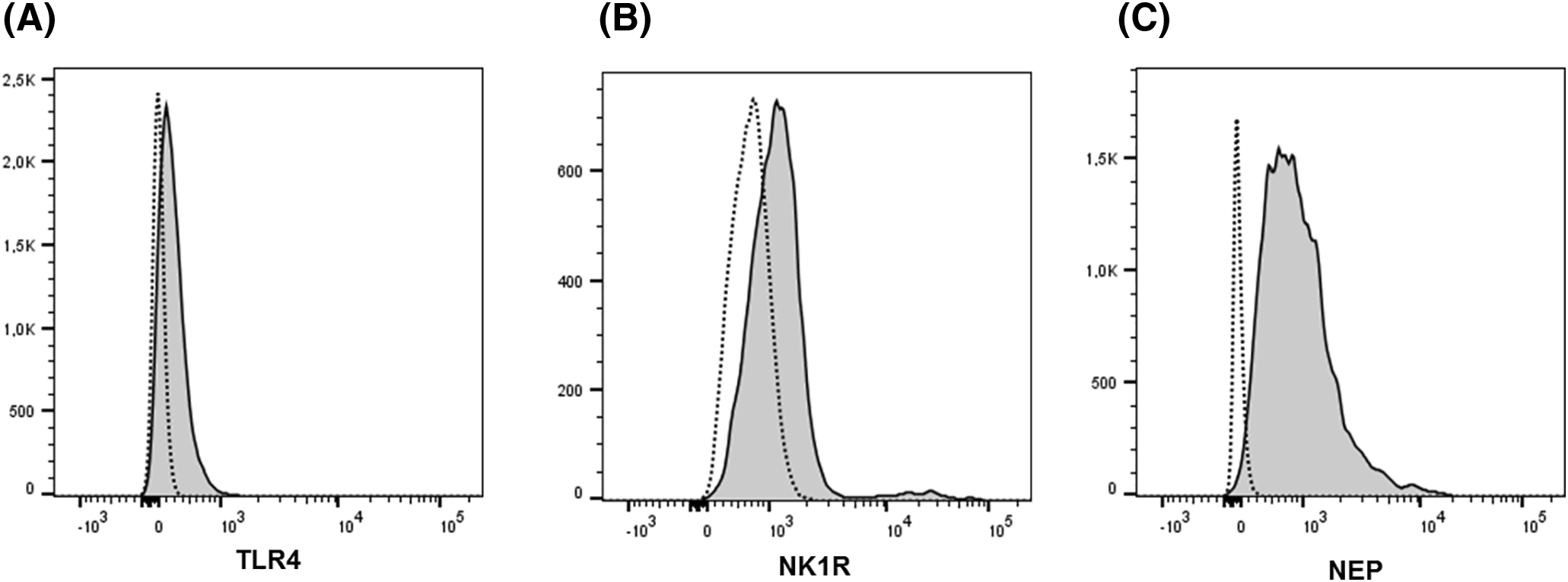

Supplement: Supplementary file 3 — Supporting Information S3 [file CLT2-11-e12009-s001.tif]
